# Case report: Greater meningeal inflammation in lumbar than in ventricular region in human bacterial meningitis

**DOI:** 10.1186/cc2972

**Published:** 2004-10-27

**Authors:** Walid Naija, Joaquim Matéo, Laurent Raskine, Jean-François Timsit, Anne-Claire Lukascewicz, Bernard George, Didier Payen, Alexandre Mebazaa

**Affiliations:** 1 Fellow, Department of Anesthesiology and Critical Care Medicine, Lariboisière University Hospital, Paris, France; 2 Attending, Department of Anesthesiology and Critical Care Medicine, Lariboisière University Hospital, Paris, France; 3 Attending, Department of Microbiology, Lariboisière University Hospital, Paris, France; 4 Professor, Medical ICU, Bichat University, Paris, France; 5 Assistant Professor, Department of Anesthesiology and Critical Care Medicine, Lariboisière University Hospital, Paris, France; 6 Professor and Chairman, Department of Neurosurgery, Lariboisière University Hospital, Paris, France; 7 Professor and Chairman, Department of Anesthesiology and Critical Care Medicine, Lariboisière University Hospital, Paris, France; 8 Professor, Department of Anesthesiology and Critical Care Medicine, Lariboisière University Hospital, Paris, France

**Keywords:** chordoma, lumbar puncture, meningitis, sepsis

## Abstract

Differences in the composition of ventricular and lumbar cerebrospinal fluid (CSF) based on single pairs of samples have previously been described. We describe a patient that developed post-surgical recurrent meningitis monitored by daily biochemical and bacteriological CSF analysis, simultaneously withdrawn from lumbar space and ventricles. A 20-year-old Caucasian man was admitted to the ICU after a resection of a chordoma that extended from the sphenoidal sinus to the anterior face of C2. CSF was continuously leaking into the pharyngeal cavity after surgery, and three episodes of recurrent meningitis, all due to *Pseudomonas aeruginosa *O12, occurred. Our case showed permanent ventricular-to-lumbar CSF gradients of leukocytes, protein and glucose that were increased during the acute phase of meningitis, with the greatest amplitude being observed when bacteria were present in both ventricular and lumbar CSF. This might suggest a greater extent of meningeal inflammation in the lumbar than in the ventricular region. Our case also showed that the increase in intravenous antibiotics (cefepim from 8 to 12 g/day and ciprofloxacine from 1.2 to 2.4 g/day) led to an increase in concentration in plasma but not in CSF.

## Introduction

Bacterial meningitis and ventriculitis remain the most frequent complication in neurosurgery. Diagnosis is based almost exclusively on biochemical and bacteriological analysis of cerebrospinal fluid (CSF) withdrawn either by puncture in the lumbar space or through an external drain located either in the lumbar or ventricular space. It is established that CSF infection is strongly suspected in the presence of a positive CSF culture and/or of a CSF : serum glucose ratio of less than 0.6 and/or of a CSF leukocyte count of more than 11/mm^3 ^in the lumbar space [1].

Differences in the composition of ventricular and lumbar CSF, based on single pairs of CSF samples, were previously described [2-4]. These studies showed a rostrocaudal gradient of leukocytes and protein and an inverse gradient of glucose in the first CSF withdrawn in patients with a confirmed diagnosis of meningitis. However, the time course of a ventricular-to-lumbar gradient of leukocytes, glucose and protein, during the occurrence and the relief of meningitis, remains unknown.

Here we describe a patient who developed, after surgery for a chordoma of the clivus, three episodes of recurrent meningitis due to *Pseudomonas aeruginosa *O12. The last two episodes were monitored by daily biochemical and bacteriological analysis of CSF withdrawn in parallel from the lumbar space and ventricles by external lumbar drainage (ELD) and external ventricular drainage (EVD).

## Case report

A 20-year-old Caucasian man with no medical history was admitted for elective surgery of a chordoma that extended from the sphenoidal sinus to the anterior face of C2. The first surgical step consisted of a subtotal removal of the tumour by a transfrontal approach. An EVD was inserted at day 1 (D1) because of the appearance of hydrocephalia.

At D10, the second approach consisted in a transoral resection of the tumour with a reconstruction of the pharyngeal wall with skin taken from the arm. However, the wall was not totally occlusive, with a continuous CSF leak into the pharyngeal cavity. Seven days later (D17), the patient developed meningitis with fever and a white blood cell count of 13,800/mm^3^. CSF withdrawn through the ventricular drain showed CSF leukocytes at 830/mm^3^, a CSF protein concentration of 0.99 g/l and a CSF glucose concentration of 3 mmol/l (for a glycaemia of 6 mmol/l). A *Ps. aeruginosa *O12 resistant to almost all antibiotics except ceftazidime and polymyxin B, similar to that repeatedly found in the oral cavity, grew in CSF culture. It was therefore decided to replace the ventricular drain with another in the controlateral hemisphere for two purposes: first, to withdraw CSF to reduce CSF leakage by the fistula, and second, to perform a biochemical and bacteriological analysis. Antibiotherapy was started with intravenous (i.v.) ceftazidime (6 g/day for 2 days, followed by 8 g/day for 25 days) combined with amikacin and polymyxin B both in the ventricles.

A clear improvement in the meningitis allowed us to perform the third and last approach (at D42): an occipito-cervical fixation procedure with EVD removed. Three days later (D45), the patient developed a new episode of hydrocephalia. It was therefore decided to introduce ELD rather than EVD.

Twelve days later (D57), the patient developed a second episode of meningitis: fever, lumbar CSF leukocytes at 14,000/mm^3^, a CSF protein concentration of 1.88 g/l and a glucose concentration of 0.9 mmol/l (for a glycaemia of 6 mmol/l). A CSF culture found the same bacteria as in the first episode of meningitis. This second episode was considered to be related to the persistent pharyngeal fistula. The ELD was replaced with a new one and EVD was added because of the suspicion of an additional obstruction in the 4th ventricle related to post-surgical oedema. Meningitis was treated with an increasing dose of i.v. ciprofloxacin (from D61 to D95: 1.2 g continuously over 24 hours for 4 days, followed by 2.4 g over 24 hours for a further 31 days) and i.v. cefepim (from D61 to D95: 4 × 2 g/day for 4 days to 4 × 3 g/day for a further 31 days; see below for the inhibitory minimal concentration and the plasma and CSF concentrations of antibiotics) and amikacine and polymyxin B colistine both administered directly into the ventricles.

The third episode of meningitis appeared at D66 with identification of the same *Ps. aeruginosa *O12 in CSF culture, increased CSF protein and decreased CSF glucose levels in both ELD and EVD. Antibiotics were kept constant and, despite negative cultures, ELD and EVD were replaced with new drains. Interestingly, since this last episode of meningitis, the pharyngeal fistula disappeared, which indicated the end of pharyngeal contamination of CSF. The patient improved rapidly and was discharged home at D108. No further episode of meningitis during the next 3 years, nor any toxic effect related to the high doses of antibiotics, was observed. It is noteworthy that repetitive cerebral computed tomography scans showed no empyema.

**Figure 1:**
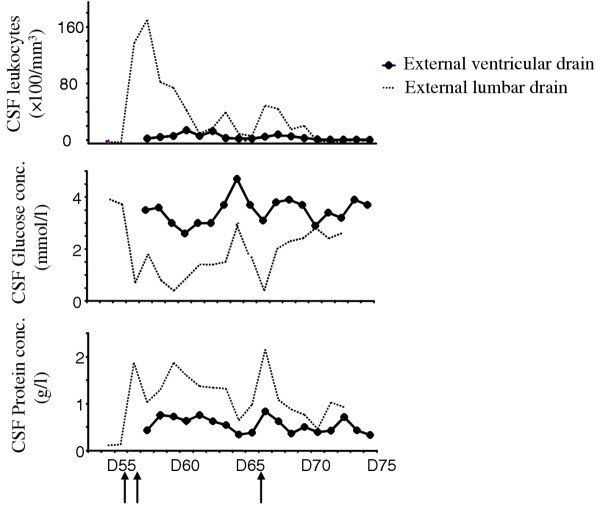
Time course of the ventricular-to-lumbar gradient of cerebrospinal fluid leukocyte, glucose and protein concentrations in cerebrospinal fluid. The arrows represent days of positive cerebrospinal fluid culture.

Figure 1 shows the time course of the following parameters: leukocyte counts, glucose and protein concentrations, measured in parallel in CSF from EVD and ELD, for 17 days (D57 to D73) corresponding to the second and third episodes of meningitis. Figure 1 shows strikingly that the leukocytes and the protein concentration were always higher and the glucose concentration was always lower in ELD than in EVD. Interestingly, the highest ventriculo-lumbar CSF gradients in leukocytes, protein and glucose concentration were present at the very acute phase of meningitis, when *Ps. aeruginosa *O12 was present in the meningeal cavity.

Our case also showed that the increase in the amount of antibiotics given did increase their concentration in plasma but not in CSF. Indeed, i.v. cefepim was increased from 8 to 12 g/day and i.v. ciprofloxacin from 1.2 to 2.4 g/day from D64 to D95. This induced a persistent increase in plasma cefepim concentration from 46 μ g/ml to more than 60 μg/ml and plasma ciprofloxacin concentration from 0.2 μg/ml to more than 1.0 μg/ml. However, only a transient increase in cefepim concentration (D63, 7 μg/ml; D73, 15 μg/ml; D81 and D95, less than 9 μg/ml) and no increase in ciprofloxacin concentration (0.4–0.5 μg/ml from D63 to 95) were seen in lumbar and ventricular CSF. It is noteworthy that the inhibitory minimal concentrations of cefepim and ciprofloxacin for *Ps. aeruginosa *O12 were 16 and 0.25 mg/ml, respectively.

## Discussion

Our case report followed ventriculo-lumbar CSF gradients in leukocytes, protein and glucose concentration during two episodes of post-operative recurrent meningitis due to *Ps. aeruginosa *O12. It showed the presence of a rostrocaudal gradient of leukocytes and protein and an inverse gradient of glucose. This confirmed previous work that showed greater leukocytes and protein concentration in lumbar than in ventricular CSF in patients with a central neural system infection, mostly after neurosurgery [2,4]. However, patients from those studies each had only one pair (ventricular and lumbar) of measurements within a 24-hour interval [2] and glucose concentration in CSF was measured in only six patients [4].

We extend previous studies by showing that the greatest amplitude of ventricular-to-lumbar gradients for all measured parameters (leukocytes, protein and glucose concentration) were seen during the very acute phase of meningitis, when bacteria were present in the meningeal cavity. The mechanisms of such ventricular-to-lumbar gradients are unknown. Our data strongly suggest a compartmentalization of meningeal inflammation in the ventricular and lumbar area. Indeed, similar bacteria, here *Ps. aeroginusa *O12, in similar quantities, seemed to induce a greater alteration of meningeal permeability with greater leukocyte and protein concentrations and a lower glucose concentration in the lumbar than the ventricular CSF region. Although still debatable, the decrease in glucose concentration in CSF seems to be less related to a 'leukocyte-induced glucose consumption' but rather to a meningeal shift of glucose metabolism to anaerobic glycolysis, as indicated by the concomitant increase in CSF lactate concentration and/or a decrease in meningeal glucose transport [5]; the latter is probably directly related to the degree of meningeal inflammation. An alternative explanation of the existence of a rostrocaudal gradient of leukocytes is that leukocytes from ventricular CSF might fall by gravity to lumbar CSF. However, as explained above, a greater concentration of leukocytes cannot by itself explain a greater protein concentration and a lower glucose concentration in lumbar CSF. Accordingly, our study suggests that meningeal inflammation was greater in the lumbar than the ventricular region in our patient with CSF infection due to a pharyngeal fistula.

Recurrent meningitis led us to increase the antibiotic dosage to achieve a better concentration in CSF [6]. Surprisingly, only a transient increase in CSF cefepim concentration and no change in CSF ciprofloxacine concentration were observed despite a more than 50% increase in plasma concentrations of both antibiotics. The transient increase in cefepim in CSF paralleled that of protein in CSF and could be related to the transient alteration in meningeal permeability.

In summary, this case report shows that the maximal rostrocaudal gradient of leukocytes, protein and glucose was seen in the very acute phase of meningitis. This strongly suggests a greater alteration in the meningeal barrier and very probably a greater meningeal inflammation in the lumbar than the ventricular regions.

## Key messages

• 	The paper describes a patient that developed, after surgery for a chordoma of the clivus, three episodes of recurrent meningitis due to *Ps. aeruginosa* O12.

• 	Episodes were monitored by biochemical and bacteriological daily analysis of CSF withdrawn in parallel from lumbar space and ventricles by external lumbar and ventricular damamge.

• 	We observed a permanent ventricular-to-lumbar CSF gradients of leukocytes, protein and glucose that increased during the acute phase of meningitis, with the greatest amplitude observed when bacteria was present in both ventricular and lumbar CSF.

• 	This may suggest a greater extent of meningeal inflammation in lumbar than in ventricular region

## Abbreviations

CSF = cerebrospinal fluid; D1, day of insertion of EVD; ELD = external lumbar drainage; EVD = external ventricular drainage; ICU = intensive care unit; i.v. = intravenous.

## Competing interests

The author(s) declare that they have no competing interests.

## Author's contributions

WN and AM coordinated the data analysis and drafted the manuscript. JM, LR and J-F T participated in bacteriological analysis. A-C L and BG participated in analysis of clinical data. DP helped to draft the manuscript. All authors read and approved the final manuscript
